# Expression of *blaA* Underlies Unexpected Ampicillin-Induced Cell Lysis of *Shewanella oneidensis*

**DOI:** 10.1371/journal.pone.0060460

**Published:** 2013-03-28

**Authors:** Jianhua Yin, Linlin Sun, Yangyang Dong, Xun Chi, Weiming Zhu, Shu-hua Qi, Haichun Gao

**Affiliations:** 1 Institute of Microbiology and College of Life Sciences, Zhejiang University, Hangzhou, Zhejiang, China; 2 School of Medicine and Pharmacy, Ocean University of China, Qingdao, Shandong, China; 3 South China Sea Institute of Oceanology, The Chinese Academy of Sciences, Guangzhou, Guangdong, China; University Medical Center Utrecht, The Netherlands

## Abstract

*Shewanella oneidensis* is a facultative anaerobic γ-proteobacterium possessing remarkably diverse respiratory capacities for reducing various organic and inorganic substrates. As a veteran research model for investigating redox transformations of environmental contaminants the bacterium is well known to be a naturally ampicillin-resistant microorganism. However, in this study we discovered that ampicillin has a significant impact on growth of *S. oneidensis*. Particularly, cell lysis occurred only with ampicillin at levels ranging from 0.49 to 6.25 µg/ml but not at 50 µg/ml. This phenotype is attributable to insufficient expression of the β-lactamase BlaA. The subsequent analysis revealed that the *blaA* gene is strongly induced by ampicillin at high (50 µg/ml), but not at low levels (2.5 µg/ml). In addition, we demonstrated that penicillin binding protein 5 (PBP5), the most abundant low molecular weight PBP (LMW PBP), is the only one relevant to β-lactam resistance under the tested conditions. This nonessential PBP, largely resembling its *Escherichia coli* counterpart in functionality, mediates expression of the *blaA* gene.

## Introduction

Since their discovery, β-lactam antibiotics have been widely used to treat bacterial infections. They mimic the D-Ala-D-Ala dipeptide in an elongated conformation and covalently modify the active site of penicillin binding proteins (PBPs), enzymes that play key roles in the peptidoglycan assembly [1]. As a result, β-lactams, as bactericidal antibiotics, disturb the balance between peptidoglycan synthesis and degradation, leading to cell lysis eventually. Although recent studies have proposed that the β-lactam-induced lysis is mediated enzymatically [2]–[4], the underlying molecular mechanisms remain poorly understood.

PBPs are classified into two groups based on their relative mobility in sodium dodecyl sulfate-polyacrylamide gel electrophoresis (SDS-PAGE): high molecular weight (HMW) and low molecular weight (LMW). In *Escherichia coli*, there are at least 12 PBPs, which differ from one another functionally [5]. HMW PBPs (PBP1a, PBP1b, PBP1c, PBP2 and PBP3) are responsible for transglycosylation and transpeptidation in peptidoglycan synthesis. Except for PBP1c, HMW PBPs are essential for cell elongation, maintenance of cellular morphology, and normal division. On the contrary, most of *E. coli* LMW PBPs, including PBP4, PBP5, PBP6, and PBP7, are DD-carboxypeptidases (DD-CPases) and/or endopeptidases that are involved in the regulation of the level of peptidoglycan reticulation, but dispensable for survival in laboratory cultures [6]–[9].

Bacteria have evolved several means to counteract β-lactams. One of the most common strategies in Gram-negative bacteria is to produce β-lactamases that hydrolyze the antibiotics. There are two major classes of β-lactamases based on their primary structure. Serine β-lactamases harbor an SXXK motif that is essential for catalytic reaction, whereas metallo-β-lactamases require one or two Zn^2+^ ions for activity by binding with His/Cys/Asp residues at the active site [10]. Another important strategy is to utilize extra PBPs with low affinity for the β-lactams, particularly LMW PBPs although many questions about the functions of these proteins remain unresolved [5], [6], [11]. *E. coli* PBP4 and PBP5, sharing a common ancestor with β-lactamases, have been shown to be able to hydrolyze penicillin *in vitro* although *in vivo* evidence is lacking [12], [13]. Recently, it has been proposed that redundant PBPs, especially PBP5 whose removal renders cells significantly more susceptible to β-lactams, may serve as traps for β-lactams, shielding over the essential PBPs from inhibition by β-lactams [8]. Intriguingly, in *Pseudomonas aeruginosa* the inactivation of PBP4 triggered overproduction of the chromosomal β-lactamase AmpC, and thus to β-lactam resistance [7].

*Shewanella oneidensis*, a Gram-negative facultative anaerobe, is renowned for its respiratory versatility [14]. Because of the potential application in bioremediation, biogeochemical circulation of minerals and bioelectricity, the bacterium has been intensively investigated, especially in the field of metal reduction and stress response [14], [15]. In recent years, *S. oneidensis* has become a research model for investigating respiratory pathways, biofilm formation, biofuel production, and bioenergy generation as well [16]–[23]. In the *Shewanella* research community, it is well known that most, if not all strains are naturally resistant to ampicillin, a widely utilized β-lactam antibiotic in genetic manipulation [24]. Surprisingly, Poirel *et al.* reported that *S. oneidensis* is susceptible to all 14 β-lactam antibiotics (excluding ampicillin) of four β-lactam classes tested [25]. Apart from this, little is known about how *S. oneidensis* cells respond to these antibiotics although the subject is relevant to their utilization for genetic screens as well as in natural environments.

Here we report that certain β-lactams induce lysis of *S. oneidensis* cells only within a narrow concentration range. We show that BlaA, one of seven putative β-lactamases encoded in the genome, is the only one conferring β-lactam resistance under the conditions tested. Insufficient expression of this β-lactamase predominantly accounts for cell lysis by low doses of ampicillin. We also found that expression of *blaA* is not only responsive to β-lactam antibiotics but also significantly affected by PBP5, the most abundant LMW PBP.

## Results

### Ampicillin and penicillin inhibit pellicle formation at sub-MIC concentrations

A natural product screen identified a penicillin-like compound to inhibit growth and pellicle (biofilm at the air-liquid interface) formation most effective at sub-inhibitory concentrations (sub-MIC) (data not shown). The finding was unexpected given that *Shewanella* is known to be naturally resistant to penicillin and ampicillin. Moreover, this discovery also implicates that some of the common clinically used antibiotics may have unexpected effects on *S. oneidensis* and likely other bacteria at concentrations other than at the therapeutic levels. To this end, we assayed pellicle formation of *S. oneidensis* in the presence of ten commonly used antibiotics.

**Figure 1:**
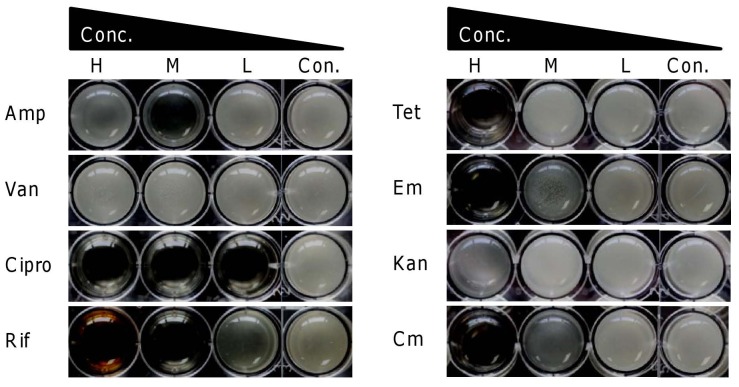
Pellicle formation of *S. oneidensis* in the presence of commonly used antibiotics (8 of 10 tested were shown). Late-exponential phase cultures (∼0.6 of OD_600_) were diluted 1∶100 with LB broth, aliquotted into 24-well plates (2 ml/well) and incubated statically at 30°C. The wells were photographed 20 h after inoculation. Concentrations (H, M, L µg/ml): ampicillin (Amp, 50, 2.5, 0.125), vancomycin (Van, 50, 2.5, 0.125), and ciprofloxacin (Cipro, 50, 2.5, 0.125), rifampicin (Rif, 50, 2.5, 0.125), tetracycline (Tet, 1.2, 0.06, 0.003), erythromycin (Em, 12.5, 0.625, 0.031), kanamycin (Kan, 5, 0.25, 0.0125), chloramphenicol (Cm, 8.5, 0.42, 0.021). In this and all other figures, Con. represents the antibiotic-free control.

As shown in Table 1 at concentrations routinely used all but ampicillin and vancomycin abolished growth and pellicle formation (Fig. 1). When these antibiotics were added at permissive concentrations, growth and pellicle formation were indistinguishable from that in the control. Notably, ciprofloxacin was extremely effective against *S. oneidensis*, preventing cell growth at 0.125 µg/ml. Interestingly, although *S. oneidensis* is resistant to both ampicillin and vancomycin, the response of cells to these two agents was different. At all concentrations, vancomycin had little impact on growth or pellicle formation, probably due to its low permeability in Gram-negative bacteria [26]. In contrast, ampicillin at the subinhibitory level of 2.5 µg/ml significantly delayed formation of pellicles, although they emerged 24 h after inoculation and eventually developed into mature ones which were identical to those formed in the absence of the agent. At 0.125 or 50 µg/ml, however, there was little or no effect.

**Table 1:** Susceptibility of *S. oneidensis* to various antibiotics.

Antibiotic	Concentration (µg/ml) of antibiotics^a^		
	Resistant	Intermediate resistant	susceptible
Ampicillin	100	ND	ND
Chloramphenicol	1	2	4
Ciprofloxacin	ND	ND	0.125
Erythromycin	1	2	4
Gentamycin	1	2	4
Kanamycin	2.5	5	10
Neomycin	2.5	5	10
Rifampicin	0.125	1	2
Tetracycline	0.125	1	2
Vancomycin	50	ND	ND

a ND, not determined.

**Figure 2:**
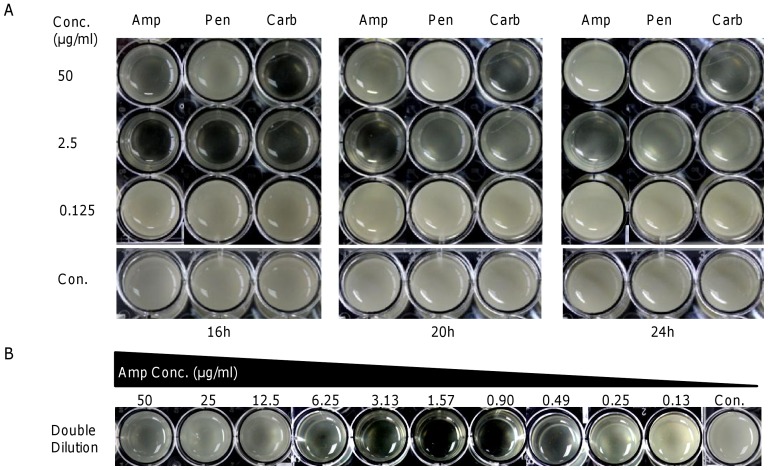
Pellicle formation of *S. oneidensis* in the presence of β-lactam antibiotics. (A) Inhibitory effects on pellicle formation were found with ampicillin and penicillin (Pen), but not carbenicillin (Carb). (B) Pellicle formation in LB broth containing ampicillin prepared by double dilution. Pellicle formation was inhibited by ampicillin at concentrations ranging from 0.49 to 6.25 µg/ml.

To examine whether the response is specific to ampicillin, we repeated the experiments with two other β-lactams, penicillin and carbenicillin (Fig. 2A). In both cases, the cells eventually overcame inhibition, grew and formed pellicles. However, there were some differences. The effect of penicillin on pellicle formation was similar to that of ampicillin, whereas carbenicillin displayed a conventional inhibitory pattern, that is, the inhibitory effect on growth and pellicle formation correlated with the antibiotic concentration. These results suggest that various β-lactams elicit different responses in *S. oneidensis*. Further analyses with other concentrations revealed that inhibition of pellicle formation by ampicillin occurred when it was added at concentrations ranging from 0.49 to 6.25 µg/ml, with 0.9–3.13 µg/ml being most effective (Fig. 2B).

### Ampicillin of sub-MIC induces cell lysis

**Figure 3:**
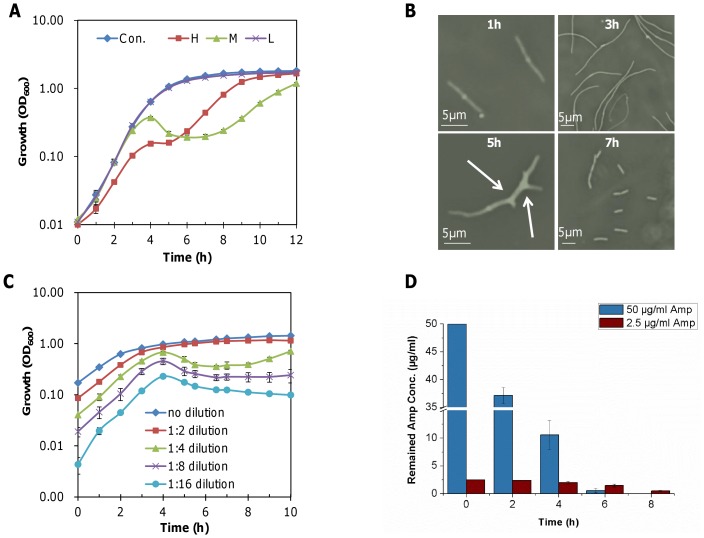
Cell lysis caused by ampicillin at 2.5 µg/ml. Cultures of late-exponential phase cells (∼0.6 of OD_600_) were diluted 1∶100 with LB broth, and incubated at 30°C in a shaker at 200 rpm. (A) Growth of *S. oneidensis* in the presence of ampicillin at H (50 µg/ml), M (2.5 µg/ml) or L (0.125 µg/ml) levels. (B) Microscopic images of cells at various times in the presence of ampicillin at 2.5 µg/ml. Arrows point to knobs and branches characterstic of treated cells. (C) Growth of cultures varying in initial cell density in the presence of ampicillin at 2.5 µg/ml. (D) Amounts of ampicillin remaining at the indicated times in cultures supplemented initially with ampicillin at 50 µg/ml or 2.5 µg/ml. In all panels, experiments were performed at least in triplicate and the error bars represent standard deviation (SD).

In the pellicle formation assay, we noticed that growth of *S. oneidensis* was delayed significantly with ampicillin at 0.49–6.25 µg/ml, implying that delayed pellicle formation may result from impaired growth and/or cell lysis. To test this hypothesis, we measured growth in shake cultures in the presence and absence of ampicillin (Fig. 3A). As expected, ampicillin at 0.125 µg/ml had no effect. In contrast, in the presence of ampicillin at 2.5 or 50 µg/ml., the optical density leveled off after 3 hours and resumed only after an extended lag. However, cultures supplemented with ampicillin at 2.5 µg/ml were distinct from those with ampicillin at 50 µg/ml in their substantially reduced optical density, an indication of cell lysis. In addition, cultures treated with 2.5 µg/ml ampicillin required about 5 hours to return to the cell density prior to lysis, in contrast to a recovery period of less than 2 hours for cultures with 50 µg/ml ampicillin. Morphologically, addition of ampicillin at these two concentrations exerted similar effects initially, resulting in formation of filamentous cells. At 50 µg/ml, filamentous cells rapidly returned to individual rods whereas in cultures with 2.5 µg/ml ampicillin, membrane knobs and blebs developed and a significant number of the cells lysed (Fig. 3B). Similar results were obtained with penicillin (Fig. S1). In contrast, cell lysis was not found with carbenicillin at all tested concentrations (Fig. S1).

The phenotype of *S. oneidensis* with ampicillin at 2.5 µg/ml resembles that of ampicillin-treated *E. coli* cells except for full recovery of growth by the former [27], [28], implying that the antibiotic may cause cell lysis by the same mechanism in these two species. As cells treated with ampicillin at 2.5 µg/ml but not 0.125 µg/ml lysed (cell density at inoculation ≤0.01 of OD_600_), we hypothesized that cells with 2.5 µg/ml ampicillin may not be able to promptly remove the antibiotic from the culture. If so, larger inocula should allow a faster removal of the antibiotic and thereby alleviate cell lysis. To test this, cells were allowed to grow to an OD_600_ of ∼0.2 without ampicillin, and this culture was then diluted by 1∶2, 1∶4, 1∶8, 1∶16 with fresh ampicillin-containing media. As shown in Fig. 3C, ampicillin at 2.5 µg/ml was able to induce cell lysis in 1∶4, 1∶8, and 1∶16 diluted cultures but not in either undiluted or 1∶2 diluted cultures, thus supporting our hypothesis. Notably, lysis occurred at the same time, 4 h after inoculation despite the difference in optical densities of these cultures.

We then asked whether removal of ampicillin can explain the phenotype of *S. oneidensis* in the presence of 50 µg/ml. Cells were grown in the presence of 2.5 and 50 µg/ml ampicillin and the amount of the remaining ampicillin was monitored over time (Fig. 3D). At 50 µg/ml of ampicillin the concentration was rapidly reduced, reaching the detection limit (∼0.5 µg/ml) within 6 h. In cultures with ampicillin at lysing concentrations, however, ampicillin remained above the threshold for 8 h. These data indicate that cell lysis is due to the slow removal of the agent from the cultures.

### β-lactamase BlaA dominates ampicillin hydrolysis in S. oneidensis

To address why cells failed to remove ampicillin when supplied at 2.5 µg/ml, we examined the genome for genes predicted to encode putative β-lactamases. In total, *S. oneidensis* possesses seven such genes, of which six reside on the chromosome (*SO0541*, *blaA*(*SO0837*), *SO0914*, *ampC*(*SO2388*), *SO3054* and *SO3474*) and one on the megaplasmid (*SOA0149*). SO0541, SO3054, SO3474 and SOA0149 belong to metallo-β-lactamases, requiring a metal ion for enzymatic activity, while AmpC and BlaA are annotated to be serine β-lactamases with substrate specificity for cephalosporins and a progenitor of carbapenem-hydrolyzing oxacillinase, respectively. The function of SO0914 is currently unknown.

**Figure 4:**
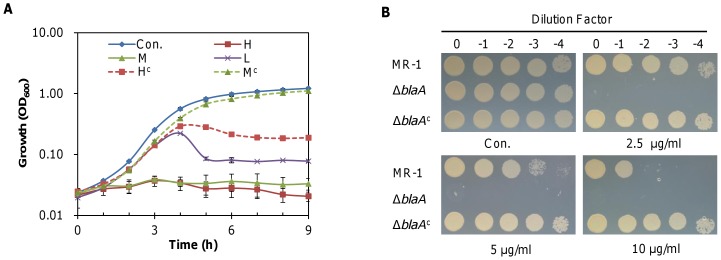
Impact of the loss of *blaA* on growth. (A) Growth of the ▵*blaA* strain in the presence of ampicillin at H (50 µg/ml), M (2.5 µg/ml) or L (0.125 µg/ml). H^c^ and M^c^ represent the ▵*blaA* strain complemented *in trans*. (B) Susceptibility assay of the ▵*blaA* strain to ampicillin. ▵*blaA* ^c^ represents the ▵*blaA* strain complemented *in trans*. Experiments were performed at least in triplicate and the error bars represent standard deviation (SD) as in (A).

We deleted each of these candidate genes individually and measured growth of the mutants in the presence of ampicillin at different levels (Fig. 4A and Fig. S2). Deletion of *SO0541*, *SO0914*, *ampC*, *SO3054*, *SO3474* and *SOA0149* resulted in a phenotype that was comparable to that of the isogenic parental strain. In contrast, loss of *blaA* substantially increased sensitivity to ampicillin, with no growth at 0.125 µg/ml. The Δ*blaA* strain failed to measurably grow when penicillin or carbenicillin at 1 µg/ml was added, whereas resistance of the other mutants to these two agents remained unaltered (Table 2). Expression of *blaA in trans* from the multiple-copy plasmid, pHG101, conferred the Δ*blaA* strain with resistance to ampicillin exceeding that of the wild type (Fig. 4A), presumably due to overproduction of BlaA [29]. In parallel, ectopic expression of *blaA* increased the MIC values of the mutant to ampicillin and prevented cell lysis (Table 2) (Fig. 4A). Moreover, similar results were obtained with the susceptibility test (Fig. 4B). These data indicate that the resistance to ampicillin can mainly be attributed to BlaA and that other putative β-lactamases are not relevant under the conditions used.

**Table 2:** MICs (µg/ml) of β-lactams for *S. oneidensis* wild type and derivative strains.

MIC (µg/ml)^a^	WT	Δ*blaA*	Δ*blaA* ^c^	Δ*SO0541*	Δ*SO0914*	Δ*ampC*	Δ*SO3054*	Δ*SO3474*	Δ*dacA*	Δ*dacA^c^*
Ampicillin	16	<1	64	16	16	16	16	16	2	16
Penicillin	32	<1	128	32	32	32	32	32	4	32
Carbenicillin	64	<1	>128	64	64	64	64	64	8	64

a MICs were recorded after 18 hours of incubation. All strains but Δ*blaA* eventually grew. Δ*blaA^c^* and Δ*dacA^c^* represent mutant strains complemented *in trans*.

### BlaA is induced by ampicillin at high concentrations

**Figure 5:**
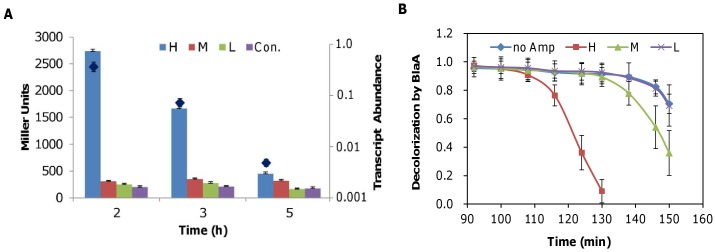
The *blaA* gene is induced by ampicillin only at high levels. Cultures of late-exponential phase cells (∼0.6 of OD_600_) were diluted 1∶100 with LB broth containing ampicillin at H (50 µg/ml), M (2.5 µg/ml), or L (0.125 µg/ml) amounts, and incubated at 30°C in a shaker at 200 rpm (A) P*_blaA_* promoter activities were determined by measuring β-galactosidase (in Miller units) using the P*_blaA_*-lacZ reporter system in the wild type. Results are averages of at least three replicates, and the error bars represent standard deviation (SD). Activity of P*_blaA_* in the presence of ampicillin at the H (50 µg/ml) level were also assayed using qRT-PCR (presented as diamonds). (B) β-lactamase activity assay. At the indicated times, samples were taken for β-lactamase activity measurements. In both panels, experiments were performed at least in triplicate and the error bars represent standard deviation (SD).

Given that BlaA is largely responsible for the resistance of *S. oneidensis* to ampicillin, we hypothesized that this β-lactamase may be induced substantially by the addition of ampicillin at high, but not low, levels. To test this, we employed a *lacZ*-reporter system to assess the promoter activity of the *blaA* gene under various conditions [30]. As shown in Fig. 5A, expression of β-galactosidase driven by the *blaA* promoter in cultures supplemented with 50 µg/ml ampicillin was almost 10 times that with 2.5 µg/ml ampicillin 2 hour after inoculation (∼0.1 of OD_600_). Transcription declined with time, coinciding with reduction of the remaining ampicillin (Fig. 3D). In contrast, expression of *lacZ* in the presence of 2.5 µg/ml ampicillin was constant and only slightly higher than that observed in cultures free of the antibiotic. Similar results were obtained with qRT-PCR when we examined expression of the *blaA* gene in samples treated with 50 µg/ml ampicillin (diamonds in Fig. 5A), confirming that *blaA* promoter is induced by ampicillin only at high concentrations.

We then measured β-lactamase activity directly using the iodometric assay [31], [32]. Penicillin instead of ampicillin was chosen as the substrate for the assay because of significant spontaneous hydrolysis of ampicillin [32]. As shown in Fig. 5B, when the antibiotic was added at 50 µg/ml, penicillin hydrolysis recorded by reduction of the optical density (decolorization) became evident about 1.5 hours after inoculation and was much more rapid than that with penicillin at 2.5 µg/ml. In contrast, penicillin at 2.5 µg/ml was not removed until 2.5 h after inoculation. In both cases, the Δ*blaA* strain was unable to hydrolyze the antibiotic (data not shown) further confirming the critical role of BlaA. As the number of cells used in these assays was comparable, these data suggest that the amount of BlaA determines resistance to ampicillin/penicillin. Overall, we conclude that cell lysis induced by ampicillin at lysing concentrations is due to the delayed removal of the antibiotic, which resulted from an insufficient amount of BlaA.

### DacA(PBP5) influences expression of BlaA in S. oneidensis

**Figure 6:**
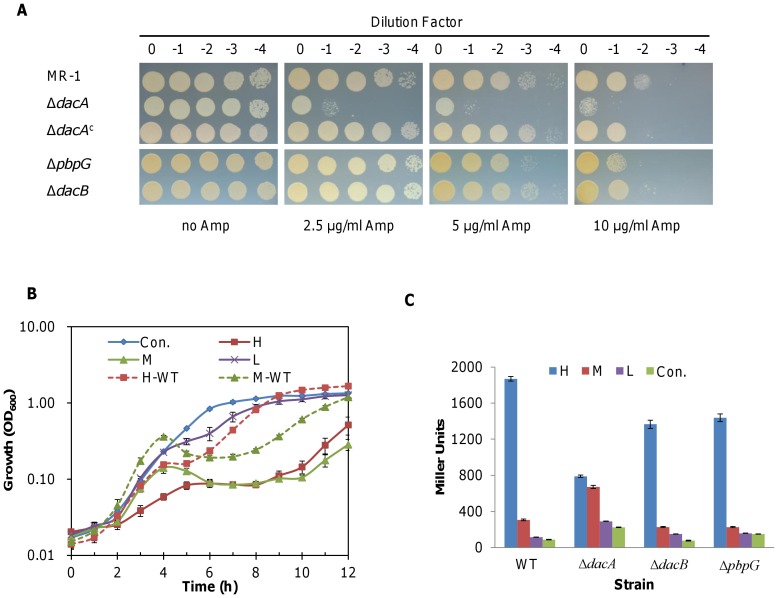
Impacts of the loss of LMW PBPs on growth in the presence of ampicillin. (A) Susceptibility assay of LMW PBP mutants ▵*dacB* (PBP4), ▵*dacA* (PBP5), and ▵*pbpG* (PBP7) to ampicillin. ▵*dacA^c^* represents the ▵*dacA* strain complemented *in trans*. (B) Growth of the ▵*dacA* strain in the presence of ampicillin at H (50 µg/ml), M (2.5 µg/ml) or L (0.125 µg/ml) levels. H-WT and M-WT represent growth of the wild type strain under the specified conditions. (C) Activities of P*_blaA-lacZ_* in strains devoid of one of the LMW PBPs. After growth for two hours, samples were taken for β-galactosidase measurements. Experiments were performed at least in triplicate and the error bars represent standard deviation (SD) as in (B and C).

PBPs are primary targets of β-lactam antibiotics and some of them are essential. It has been proposed that functionally redundant LMW PBPs, particularly PBP5 which is the most abundant, may behave as 'β-lactam traps' to protect essential ones [8]. According to the genome annotation, *S. oneidensis* has at least three LMW PBPs: DacB (SO2394, PBP4), DacA-1 (SO1164, PBP5), and PbpG (SO0999, PBP7). While DacB is predicted to be a bifunctional (DD-CPase and endopeptidase) enzyme, both DacA and PbpG are mono-functional (DD-CPase and endopeptidase, respectively). Given that the genome encodes only one PBP5, we renamed the gene *dacA-1* as *dacA*. Using the plate sensitivity assay, we found that removal of *dacA* resulted in increased susceptibility to ampicillin, consistent with findings in *E. coli* [8] (Fig. 6A, Table 2). Moreover, growth of the Δ*dacA* strain was sensitive to ampicillin even at 0.125 µg/ml and required a much longer time to resume growth with higher concentrations of ampicillin (Fig. 6B). In contrast, loss of *dacB* and *pbpG* did not result in a noticeable phenotypic change compared to the wild type (Fig. 6A).

While a comprehensive investigation of all of the possible roles that PBP5 plays was not undertaken as part of this study, we intended to determine whether PBP5 influenced expression of *blaA*. To this end, we measured the expression of the P*_blaA_*-*lacZ* fusion in the Δ*dacA* strain as well as in strains devoid of one of the other LMW PBPs in the absence and presence of ampicillin (Fig. 6C). Deletion of *dacB* (PBP4) and *pbpG* (PBP7) resulted in expression of *blaA* that was comparable to the wild type under all conditions. In the case of the Δ*dacA* strain (PBP5), however, activity of P*_blaA_* was induced by ampicillin at 2.5 µg/ml, producing β-galactosidase two to three times higher than in the wild type. Additionally, P*_blaA_* activity in the absence of ampicillin, albeit low, was higher than in the wild type, Δ*dacB* (PBP4) or Δ*pbpG* (PBP7) strains indicating that the lack of PBP5 enhanced transcription of *blaA* under these conditions. Surprisingly, in the presence of 50 µg/ml ampicillin, P*_blaA_* activity was about 50% of the wild type suggesting that the loss of PBP5 compromises induction of the *blaA* gene at the higher concentrations. While this observation supports the extended time for growth recovery of the Δ*dacA* strain in the presence of 50 µg/ml ampicillin (Fig. 6B), the underlying mechanism remains to be determined.

## Discussion

*Shewanella* contain a reservoir of antibiotic resistance determinants, especially for β-lactam antibiotics [25], [33]–[36]. In particular, *S. oneidensis* possesses seven genes predicted to encode β-lactamases, including BlaA, also named as OXA-54, an Ambler class D β-lactamase [25], [37]. BlaA, along with two analogues in other members of the *Shewanella* genus, *S. algae* and *S. xiamenensis*, has been shown to be capable of hydrolyzing carbapenem and imipenem [33], [36]. Heterogeneous expression of any of these β-lactamases in *E. coli* elevated the corresponding MICs for amoxicillin, ticarcillin, and piperacillin to at least 256 µg/ml, indicating that they were functional [25], [33], [36]. However, it has been assumed that BlaA has a dispensable role in the resistance of *S. oneidensis* to β-lactam antibiotics because of its extremely low basal expression and weak induction by imipenem and cefoxitin at subinhibitory concentrations (0.5–4 µg/ml) [25].

In this study, however, we have identified a previously undescribed phenomenon that certain β-lactams at modest concentrations delay growth and induce cell lysis. We have shown that resistance of *S. oneidensis* to β-lactams is due to BlaA. In its absence, typical β-lactams are as potent as ciprofloxacin, the most effective antibiotic against *Shewanella* tested to date. We then presented evidence that the growth inhibition and cell lysis by sub-MIC ampicillin is largely due to low expression of *blaA*, resulting in a slow removal of the antibiotic. This observation is consistent with OXA-type β-lactamases from *P. aeruginosa* which are not inducible by imipenem and cefoxitin at subinhibitory concentrations (0.2–1 µg/ml), suggesting that this group of β-lactamases may be regulated by similar mechanisms [25], [38].

Prompt and substantial production of β-lactamases is a metabolically costly endeavor for growing bacteria, but necessary for survival when high concentrations of β-lactam antibiotics are encountered. It is therefore not surprising that cells increase production of BlaA extensively when 50 µg/ml ampicillin was added. However, *Shewanella* are mainly found in marine and freshwater environments, where the concentrations of antibiotics are presumably far lower than those used therapeutically [39]. Thus, a basal level constitutive production of BlaA is needed to deal with β-lactam antibiotics at low concentrations. Apparently, there is a balance between survival and lysis as a large percentage of the cells die in the presence of sub-MIC ampicillin although the population eventually survives.

In addition to β-lactamases, redundant LMW PBPs have an important role in bacterial resistance to β-lactam antibiotics [5]. As shown here, PBP5 is required for maximum resistance to β-lactam antibiotics whereas the importance of other LMW PBPs is not evident in contrast to what has been reported for *E. coli* [8]. It has been proposed that PBP5 of *E. coli* is utilized to form a complex with ampicillin, thereby protecting essential PBPs [8]. In *Streptomyces cacaoi*, the production of β-lactamase, BlaL, is controlled by two regulators, a LysR-type activator and a PBP protein, BlaB [40]. In *P. aeruginosa*, inactivation of a nonessential PBP leads to overproduction of the chromosomal β-lactamase, AmpC, and the activation of the CreBC two-component system, a major regulator involved in β-lactam resistance [7]. These findings are consistent with our results that removal of *S. oneidensis* PBP5 resulted in enhanced expression of *blaA* in the absence of ampicillin, implying that nonessential PBPs may have a general role in linking β-lactam sensing and β-lactamase production. However, the mechanisms by which these nonessential PBPs exert their regulatory roles appear to be more complex as loss of PBP5 reduces expression of BlaA in response to 50 µg/ml ampicillin, indicating that PBP5 is required for effective and robust response to certain β-lactam antibiotics. Because of its abundant expression at the early exponential phase, PBP5 is a good candidate for a rapid response to protect vulnerable early exponential cells from these β-lactam antibiotics [41].

An important challenge for the future will be to determine how expression of *blaA* responds to β-lactam antibiotics and how PBP5 mediates this process in *S. oneidensis*. PBP5 localizes to the lateral envelope and at septal constrictions. It lacks any DNA-binding domain, ruling out a direct regulatory role at the transcription level [11]. A possible mechanism is that depletion of PBP5 by the binding of β-lactam antibiotics triggers the production of β-lactamases. Another possibility is that expression of β-lactamase is mediated by certain peptidoglycan fragments resulting from peptidoglycan turnover [42]. As PBP5 regulates the number and kinds of possible peptide crosslinks in peptidoglycan, we would anticipate that loss of PBP5 could result in significant changes in the array of peptidoglycan fragments produced during peptidoglycan synthesis [11]. One or more of these may serve as the signal to eventually alter expression of the *blaA* gene.

## Methods

### Bacterial strains, plasmids and culture conditions

Bacterial strains and plasmids are listed in Table 3 [29], [30]. *S. oneidensis* and *E. coli* were cultivated aerobically in Luria-Bertani (LB) medium at 30°C and 37°C, respectively. Unless otherwise specified, for genetic manipulation antibiotics were used at the following concentrations: ampicillin at 100 µg/ml, kanamycin at 50 µg/ml, and gentamycin at 15 µg/ml.

**Table 3:** Bacterial strains and plasmids used in this study.

Strain or plasmid	Description	Reference or source
*E. coli* strains		
DH5α	Host for regular cloning	Lab stock
WM3064	Donor strain for conjugation; Δ*dapA*	W. Metcalf, UIUC

*S. oneidensis* strains		
MR-1	Wild type	Lab stock
HG0541	*SO0541* in-frame mutant derived from MR-1; Δ*SO0541*	This study
HG0837	*blaA* in-frame mutant derived from MR-1; Δ*blaA*	This study
HG0914	*SO0914* in-frame mutant derived from MR-1; Δ*SO0914*	This study
HG0999	*pbpG* in-frame mutant derived from MR-1; Δ*pbpG*	This study
HG1164	*dacB* in-frame mutant derived from MR-1; Δ*dacB*	This study
HG2388	*ampC* in-frame mutant derived from MR-1; Δ*ampC*	This study
HG2394	*dacA* in-frame mutant derived from MR-1; Δ*dacA*	This study
HG3054	*SO3054* in-frame mutant derived from MR-1; Δ*SO3054*	This study
HG3474	*SO3474* in-frame mutant derived from MR-1; Δ*SO3474*	This study
HGA0149	*SOA0149* in-frame mutant derived from MR-1; Δ*SOA0149*	This study

Plasmids		
pDS3.0	Amp^r^, Gm^r^, derivative from suicide vector pCVD442	Lab stock
pHG101	Promoterless broad host Km^r^ vector used for complementation	[29]
pHG102	pHG101 containing the *arcA* promoter	[29]
pTP327	Ap^r^, Tet^r^, Broad host *lacZ* reporter vector	[30]
pTP327-P*_blaA_*	pTP327 containing 400 bp upstream sequence of *blaA*	This study
pTP327-P*_dacB_*	pTP327 containing 400 bp upstream sequence of *dacB*	This study

### Construction and complementation of in-frame deletion mutants

In-frame deletion mutants were constructed using the fusion PCR method was as previously described [43]. Primers used in this study are listed in Table S1. Each deletion mutation was verified by sequencing of the mutated region.

For genetic complementation, either promoterless pHG101 or its derivative pHG102, which contains the *S. oneidensis arcA* promoter for genes not in proximity to their promoter, was used [29]. Introduction of each verified complementation vector into the corresponding mutant was achieved by mating with *E. coli* WM3064 containing the vector, and confirmed by plasmid extraction, restriction enzyme mapping and sequencing.

### Growth and pellicle formation of S. oneidensis

Pellicle formation of *S. oneidensis* was achieved essentially as described previously [23]. In brief, cultures grown to the late-exponential phase (∼0.6 of OD_600_) were used as initiation seeding cultures (ISC) to prepare the starting cultures for various experiments. For growth measurement and pellicle formation, the starting cultures were prepared by a 1∶100 dilution of ISC with fresh LB broth. Cultures were incubated at 30°C in an incubator shaker at 200 rpm. For pellicle formation, the diluted cultures were aliquotted into 24-well plates with a volume of 2 ml per well. Antibiotics and natural products were added to each well at three concentrations. The plates were kept at the room temperature for observation. The morphology of cells was examined with a Motic BA310 phase-contrast microscope. Micrographs were captured with a Moticam 2306 charged-coupled-device camera and Motic images advanced 3.2 software. All experiments were conducted at least in triplicate.

### Antibiotic susceptibility assay

Antibiotic susceptibility of *S. oneidensis* was determined with both liquid and solid cultures. For antibiotics commonly used in genetic manipulation, the highest concentrations were set according to the molecular biology manual and lower concentrations were prepared by double dilution. Three µl of ISC cultures were spotted onto LB agar plates containing antibiotics of varying concentrations. The plates were incubated for up to 3 days and scored for growth each day. No growth, some growth after 3 days, and full growth after 1 day were considered susceptible, intermediate resistant, and resistant, respectively. Susceptibility assays on plates were also used to compare differences in ampicillin resistance among *S. oneidensis* strains. In this case, ISC cultures were used to prepare a decimal dilution series. Three µl of each dilution was placed onto LB plates supplemented with antibiotics at different concentrations. The plates were incubated for 18 hours at 30°C and then photographed.

Liquid cultures were utilized to determine the minimum inhibitory concentration (MIC). The starting cultures were prepared by a 1∶100 dilution of ISC with fresh LB medium supplemented with the antibiotics of interest. The cultures were incubated as described above. The MIC for a given agent was recorded as the lowest concentration that completely inhibited growth in 18 h.

### β-galactosidase activity assay

To determine the activity of the various promoters, the sequences of target promoters (∼400 bp) were amplified and cloned into the transcriptional fusion vector, pTP327, using restriction sites within primers as listed in Table S1 [30]. The resulting transcriptional fusion vector was transformed into *E. coli* WM3064, verified by sequencing, and transferred into *S. oneidensis* strains by conjugation. Cells at various growth phases (30°C) were harvested by centrifugation at 4°C, washed with PBS (phosphate buffered saline), and treated with lysis buffer (0.25 M Tris/HCl, (pH 7.5), 0.5% Trion-X100). The protein concentration of the cell lysates was determined using a Bradford assay with BSA as a standard (Bio-Rad). β-Galactosidase activity assays were performed using an assay kit (Beyotime, China) according to manufacturer's instructions as described previously [29]. Activity is expressed in Miller units [44].

### β-lactamase activity assay

β-lactamase activity was determined using the iodometric method as described elsewhere [31], [32]. Cells at the late-exponential phase (∼0.6 of OD_600_) were harvested by centrifugation at 4°C washed with PBS (phosphate buffered saline). The optical density (OD_620_) of the reaction mix was recorded over time.

### Quantitative RT-PCR (qRT-PCR) analysis

Quantitative real-time reverse transcription-PCR (qRT-PCR) analysis was carried out with an ABI7300 96-well qRT-PCR system (Applied Biosystems) essentially as described previously [45]. The expression of each gene was determined from three replicas in a single real-time qRT-PCR experiment. The Cycle threshold (*C_T_*) values for each gene of interest were averaged and normalized against the *C_T_* value of 16s rRNA, whose abundance was constant during exponential phase. The relative abundance (RA) of each gene compared to that of 16s rRNA was calculated using the equation RA = 2^−*ΔCT*^.

### Chemical assays

Culture supernatants were subjected to High-performance liquid chromatography (HPLC) analysis for determination of the ampicillin concentrations essentially as previously described [46]. Cell cultures were filtered through a hydrophilic 0.2 µm filter (Millipore, USA). Acetonitrile and chloroform were added to precipitate proteins and remove lipid-soluble components, respectively REF??. Aliquots (10 µL) of the final supernatants were injected automatically into an HPLC (Agilent 1200, USA) with a reverse-phase C18 column (150 mm×4.6 mm; 5 µm, 100 A; Phenomenex, Germany). The effluent was monitored using a UV detector at 220 nm. Standard curves were made each time employing commercial ampicillin (Sigma, USA).

## Supporting Information

Figure S1 **Growth of** ***S. oneidensis*** **cultures.** In the presence of penicillin (A) or carbenicillin (B) at H (50 µg/ml), M (2.5 µg/ml) or L (0.125 µg/ml) levels.
(PDF)

Figure S2 **Ampicillin susceptibility assay for various strains, in which one of predicted β-lactamases was deleted.** Three-microliter cultures of the late-exponential phase (∼0.6 of OD600) were dropped on LB agar plates supplemented with ampicillin varying in concentrations. Plates were incubated at 30°C and results were photographed at18 h.
(PDF)

Table S1 Primers used in this study.
(PDF)
