# Supplementary material for: Expression of blaA Underlies Unexpected Ampicillin-Induced Cell Lysis of Shewanella oneidensis
Source: PLoS One. 2013 Mar 28;8(3):e60460. doi: 10.1371/journal.pone.0060460 (PMC3610667; doi:10.1371/journal.pone.0060460)
Supplement: Figure S1 — Growth of S. oneidensis cultures. In the presence of penicillin (A) or carbenicillin (B) at H (50 µg/ml), M (2.5 µg/ml) or L (0.125 µg/ml) levels. (PDF) [file pone.0060460.s001.pdf]

Figure S1

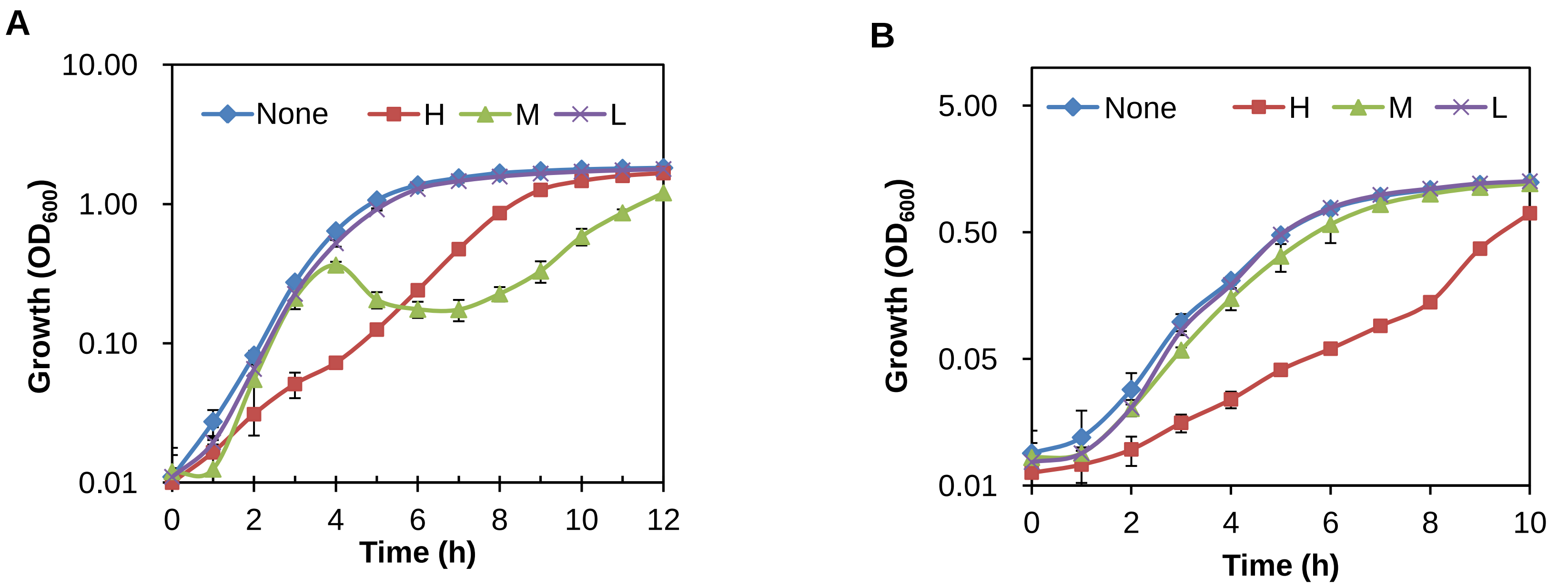

FIG. S1. Growth of *S. oneidensis* cultures in the presence of penicillin (A) or carbenicillin (B) at H (50 µg/ml), M (2.5 µg/ml) or L (0.125 µg/ml) levels.
