# Supplementary material for: Expression of blaA Underlies Unexpected Ampicillin-Induced Cell Lysis of Shewanella oneidensis
Source: PLoS One. 2013 Mar 28;8(3):e60460. doi: 10.1371/journal.pone.0060460 (PMC3610667; doi:10.1371/journal.pone.0060460)
Supplement: Figure S2 — Ampicillin susceptibility assay for various strains, in which one of predicted β-lactamases was deleted. Three-microliter cultures of the late-exponential phase (∼0.6 of OD600) were dropped on LB agar plates supplemented with ampicillin varying in concentrations. Plates were incubated at 30°C and results were photographed at18 h. (PDF) [file pone.0060460.s002.pdf]

# Figure S2

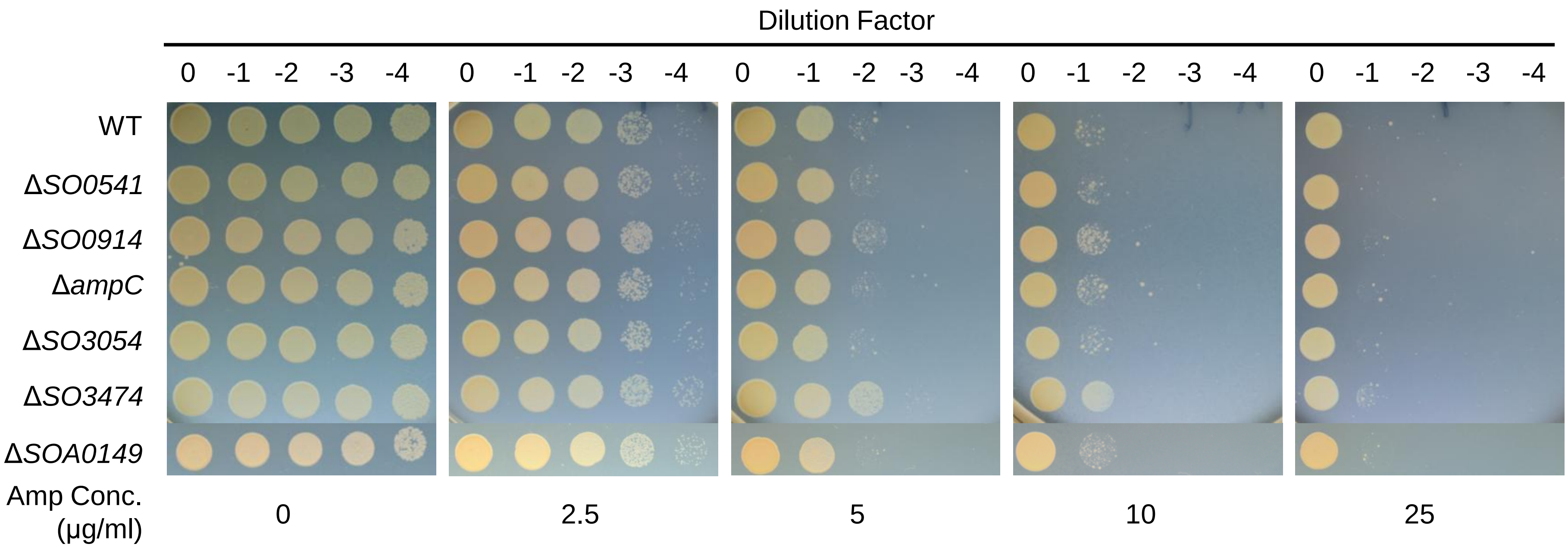


FIG. S2. Ampicillin susceptibility assay for various strains, in which one of predicted β-lactamases was deleted. Three-microliter cultures of the late-exponential phase (~0.6 of $OD_{600}$) were dropped on LB agar plates supplemented with ampicillin varying in concentrations. Plates were incubated at 30$^{o}$C and results were photographed at18 h.
