# Supplementary material for: Expression of blaA Underlies Unexpected Ampicillin-Induced Cell Lysis of Shewanella oneidensis
Source: PLoS One. 2013 Mar 28;8(3):e60460. doi: 10.1371/journal.pone.0060460 (PMC3610667; doi:10.1371/journal.pone.0060460)
Supplement: Table S1 — Primers used in this study. (PDF) [file pone.0060460.s003.pdf]

TABLE S1. Primers used in this study

**Mutagenesis**	
SO0541-5O	GAGCTCGGCGAAGAATGTCCACGGCTC
SO0541-5I	AGTTAGCACCCACATTTGGCATGGCTTAGCACAACGGCGA
SO0541-3I	GCCAAATGTGGGTGCTAACTACCTAAAGCGCTCGCCATCG
SO0541-3O	CTCGAGGGTATTTCGCGGTGGCATGGT
SO0837-5O	GAGCTCGCGATTGACCACTTCCGCCAT
SO0837-5I	ATAGTCCGGGAACTGTATGCAAGCACTATCACACCCTGCGCT
SO0837-3I	GCATACAGTTCCCGGACTATCGCTAAAACCGGATACTCGACC
SO0837-3O	CTCGAGGTCACGCTCTGCACCGATTT
SO0914-5O	GAGCTCATCCAGACGAGCTAACTCAGGCT
SO0914-5I	AAGGTACTGGAAGCTACTGCCTGGCTGCCTTGCACGCTCT
SO0914-3I	GCAGTAGCTTCCAGTACCTTTCGCGCTCGAGTATTTGAACTG
SO0914-3O	CTCGAGACTGAGCAAAGCCTTGCCGC
SO0999-5O	GAGCTCATTCCCGCACTGGATCGCCA
SO0999-5I	ATTCATGTGCCAGTGCCGTGGGCCGCAACTTCTTGCTTCG
SO0999-3I	TAAGTACACGGTCACGGCACGGCCGATGCCAACCGTTTA
SO0999-3O	CTCGAGGCACCGCCAGTAACAGCGA
SO1164-5O	GAGCTCCATTAACGATCGCGGCCCCT
SO1164-5I	ATTCGATGGGAACGATCCGCCATCAAGACGTAAGCCTTGGCG
SO1164-3I	GCGGATCGTTCCCATCGAATCGTGGGCCGCTTATACTTCCAG
SO1164-3O	CTCGAGATCCACAGCTCGTCTGGGCTAT
SO2388-5O	GAGCTCAAACGCGTGGGTATCGGGGAT
SO2388-5I	AATGACGAGGAAGTTGCTCCGGTGGCATGCACCATCAATGGT
SO2388-3I	GGAGCAACTTCCTCGTCATTGACTGGCTCAACTGGCGGTTT
SO2388-3O	CTCGAGCGGCTAAGCCTTGGGCAATCA
SO2394-5O	GAGCTCAATCAACTGAGGCTCAAAGG
SO2394-5I	CCGCATACCGAATTGCTATAGAACGAGGTAAGGTGAATAATCG
SO2394-3I	TATAGCAATTCGGTATGCGGCCACAGTTGAACAGCAAGC
SO2394-3O	CTCGAGCAAGCCATTGACTTCCTCG
SO3054-5O	GAGCTCGTCCTGTGCCCCGTTGATCG
SO3054-5I	AATCTTACGGAATCGTCTGCACGGTCGACATTCCCTCCCGGA
SO3054-3I	GCAGACGATTCCGTAAGATTCAATCCCTTTGTGGCGGATCAACT

SO3054-3O	CTCGAGGGTAGCGAATGTTGGCCGCA
SO3474-5O	GAGCTCAATAGGGGCGCACAACCGTG
SO3474-5I	GTCAGAGTCATCACCTATTGGCATCATTTCATGCTCGCCGC
SO3474-3I	CAATAGGTGATGACTCTGACCCCACGGATGCAGGGCTTG
SO3474-3O	CTCGAGGCCCGTGATGCCAAAATGTGC
SO0129A-5O	GAGCTCAAGGCATTGAAAGCCAGTGT
SO0129A-5I	GCCGGAATCCAAGATACCGACTCTGGCCTACTGTCTAAACG
SO0129A-3I	TCGGTATCTTGGATTCCGGCGTACACAGGACATTGCACAG
SO0129A-3O	CTCGAGAAACGCCTCGACATAACCTC
**Complementation**	
SO0837-CF	GGAATTCGCGATTGACCACTTCCGCCA
SO0837-CR	CCGCTCGAGTTACGCTAGGCCTTTGACGCT
SO0837-CF-arcA-I	GGAATTCATAAGCAAGGGGATATTATGCGTG
SO0837-CF-arcA-II	GGAATTCAAGCAAGGGGATATAGATGCGTG
SO1164-CF	GGAATTCCACGGGCACGGCTCGGATTA
SO1164-CR	CCGCTCGAGTTTGAAGGGAAAGGCGCAGGG
**Promoter activity**	
SO0837-5P	CCGGAATTCTACCCTCGGAAGATATTAGC
SO0837-3P	CGCGGATCCGTAATCATGGTCATAATATCCCCTTGCTTAATGC
SO1164-5P	CCGGAATTCGCCTCTAAGGATAAACAACGA
SO1164-3P	CGCGGATCCGTAATCATGGTCATCATTAACTTAGCACGTCTTTTTG
**qRT-PCR**	
SO0837-qF	ACTCGGTCGTGCCCGTGTATCA
SO0837-qR	ATCCGAATGCCGCCATCGAGC
SO1164-qF	ACCGCGGGATCATCATCCAATCA
SO1164-qR	TTCATCATGTCGACGAAAGCGCC
